# ASSESSING THE INDIVIDUAL NEEDS AND GOALS OF DEMENTIA CAREGIVERS: DEVELOPMENT OF THE COPE-ING MEASURE

**DOI:** 10.1093/geroni/igae098.2350

**Published:** 2024-12-31

**Authors:** Alyssa Aguirre, Jared Benge, Andrew Samson

**Affiliations:** The University of Texas Austin, Austin, Texas, United States; The University of Texas Austin, Austin, Texas, United States; The University of Texas Austin, Austin, Texas, United States

## Abstract

Caregivers of persons living with dementia (PLWD) are diverse, yet there is a shortage of measures that efficiently identify the personalized needs and goals of a given caregiver. To address this gap, this study outlines the development of an Individualized Needs and Goals assessment for use with the Caregiver Outcomes of Psychotherapy Evaluation (COPE-ING). In phase 1, 52 participants (N=28 caregivers; N=24 dementia professionals) were recruited to provide feedback on the preliminary COPE-ING measure. Participants rated each item on a Likert scale (1- Not important at all to 5-Extremely Important) on how important they felt each item is to dementia caregivers. While all items were endorsed as important, qualitative feedback emphasized the need for simpler language, substituting ‘cognitive impairment’ with ‘dementia’ and providing specific examples of services. A revised version of the COPE-ING was subsequently administered in an ongoing clinical sample. In preliminary findings, initial analysis revealed great heterogeneity in caregiver perceived top priorities for care, with 30% selecting learning more about dementia as a top priority, but a wide variety of topics being selected as top priorities for the remainder of the sample. These findings outline the initial development of the COPE-ING, a novel measure designed to capture the individualized needs and goals of dementia caregivers. This study underscores the importance of creating caregiver measures that extend beyond burden. The COPE-ING holds promise for use in evidence-based interventions to facilitate the rapid identification of caregiver needs and goals in hospital or community settings.

